# Construction and characterisation of glycoprotein E and glycoprotein I deficient mutants of Australian strains of infectious laryngotracheitis virus using traditional and CRISPR/Cas9-assisted homologous recombination techniques

**DOI:** 10.1007/s11262-022-01933-5

**Published:** 2022-09-20

**Authors:** Marzieh Armat, Paola K. Vaz, Glenn F. Browning, Amir H. Noormohammadi, Carol A. Hartley, Joanne M. Devlin

**Affiliations:** 1 Asia-Pacific Centre for Animal Health, Melbourne Veterinary School, Faculty of Veterinary and Agricultural Sciences, The University of Melbourne, Parkville, VIC Australia; 2 Asia-Pacific Centre for Animal Health, Melbourne Veterinary School, Faculty of Veterinary and Agricultural Sciences, The University of Melbourne, Werribee, VIC Australia

**Keywords:** CRISPR/Cas9, Glycoprotein E, Glycoprotein I, Homologous recombination, Infectious laryngotracheitis virus

## Abstract

In alphaherpesviruses, glycoproteins E and I (gE and gI, respectively) form a heterodimer that facilitates cell-to-cell spread of virus. Using traditional homologous recombination techniques, as well as CRISPR/Cas9-assisted homologous recombination, we separately deleted gE and gI coding sequences from an Australian field strain (CSW-1) and a vaccine strain (A20) of infectious laryngotracheitis virus (ILTV) and replaced each coding sequence with sequence encoding green fluorescent protein (GFP). Virus mutants in which gE and gI gene sequences had been replaced with GFP were identified by fluorescence microscopy but were unable to be propagated separately from the wildtype virus in either primary chicken cells or the LMH continuous chicken cell line. These findings build on findings from a previous study of CSW-1 ILTV in which a double deletion mutant of gE and gI could not be propagated separately from wildtype virus and produced an in vivo phenotype of single-infected cells with no cell-to-cell spread observed. Taken together these studies suggest that both the gE and gI genes have a significant role in cell-to-cell spread in both CSW-1 and A20 strains of ILTV. The CRISPR/Cas9-assisted deletion of genes from the ILTV genome described in this study adds this virus to a growing list of viruses to which this approach has been used to study viral gene function.

**Supplementary Information:**

The online version contains supplementary material available at 10.1007/s11262-022-01933-5.

The *Herpesviridae* are a family of viruses with large double-stranded linear DNA genomes encoding between 70 and 170 genes. *Gallid alphaherpesvirus*1 (infectious laryngotracheitis, ILTV) is an alphaherpesvirus that causes respiratory tract infections in chickens. It causes economic loss in poultry industries worldwide [1]. The ILTV genome is approximately 150 kb in length, with a unique long (U_L_) and a unique short sequence (U_s_). The latter is flanked by inverted repeat (IR) and terminal repeat (TR) regions.

In alphaherpesviruses, glycoproteins E and I (gE and gI, respectively) are conserved glycoproteins that play a role in viral cell-to-cell spread and virulence *in viv*o [2–6]. They form a non-covalently bound heterodimer and are not essential for replication in vitro in most alphaherpesvirus species [7, 8]. In two alphaherpesviruses, Marek’s disease virus (MDV) and varicella zoster virus (VZV), gE and gI are essential for replication in vitro [4, 9–11]. In cell culture these viruses are highly cell associated, with little extracelluar virus produced, and either do not contain a glycoprotein D (gD) gene (VZV), or have a gD gene that is not expressed in cultured cells (MDV) [12, 13]. The essentiality of gE and gI in these viruses that lack a functional gD suggests an overlapping function between gE/gI and gD [14] [9, 11].

The roles of ILTV gI and gE have been studied previously [15, 16]. Initial studies created a double gI and gE deletion mutant of the Australian field strain CSW-1 and showed that the mutant virus was unable to spread from cell-to-cell [15]. Subsequently single and double gI and gE deletion mutants of the virulent ILTV strain A489 were generated and characterised. In cell culture these mutants exhibited reduced cell-to-cell spread, but not to the same extent as the CSW-1 mutant virus [16]. To further clarify the role of ILTV gI and gE, this study aimed to generate and characterise single gI and gE deletion mutants of the Australian CSW-1 field strain, and of the Australian A20 vaccine strain.

## Materials and methods

### Cells and viruses

The gE deleted (ΔgE) and gI deleted (ΔgI) ILTV mutants were generated from the virulent Australian ILTV field strain, CSW-1 [17] and the attenuated Australian vaccine strain, A20 (Batch 1571112A, Zoetis). Primary chicken embryo kidney (CEK) cells and a continuous chicken hepatoma cell line (LMH cells) were used for virus propagation and characterisation [18]. Cells were cultured in Dulbecco’s Minimal Essential Medium (DMEM, Sigma) with 10% v/v fetal bovine serum (FBS, Sigma-Aldrich) and 50 µg ampicillin/ml. Cells were incubated in 5% CO2 in air at 37 °C.

### Construction of gene deleted ILTV

The ΔgE and ΔgI ILTV mutants were constructed using homologous recombination as described previously [15] or generated using CRISPR/Cas9-assisted homologous recombination in an ILTV infection/transfection system [19].

**Fig. 1:**
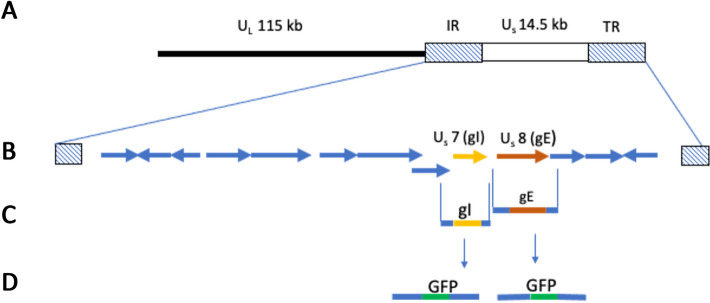
Schematic representation of ΔgE and ΔgI ILTV generation using SOE PCR and homologous recombination. **A** ILTV genome showing unique short (US), unique long (UL), terminal repeat (TR) and internal repeat (IR) regions. **B** Map of US genes including the gI and gE genes. **C** ILTV genome regions constructed to contain just the gI or gE coding sequence and the corresponding upstream and downstream regions of the ILTV genome. **D** Linearised repair plasmids in which the gI or gE coding sequence in C (above) were replaced with GFP. These plasmids underwent recombination with ILTV genomic DNA to generate ΔgE and ΔgI ILTV mutants

Briefly, DNA products containing the gene encoding enhanced green fluorescent protein (eGFP) flanked by the CSW-1 sequence extending 928 bp upstream and 989 bp downstream of gE, or extending 1051 bp upstream and 984 bp downstream of gI, were assembled using splicing by overlap extension PCR (SOE PCR) (Fig. 1) [20] using the primers listed in Table 1. The final SOE PCR products were then ligated into pGEM-T (Promega) and subsequently transformed into electrocompetent *E coli*. Following extraction (Midiprep plasmid extraction kit, Qiagen) plasmids were linearized using restriction endonucleases prior to their use in transfection experiments.

**Table 1:** Primers used in this study

PCR product	Orientation	Sequence (5'-3')*
Generation of ΔgE ILTV		
Upstream of gE	Forward	GCCTCGAACAGACTAACCG
	Reverse	TCGCCCTTGCTCACCAT**CAAGCCTACTCAACA**
Downstream of gE	Forward	TCCACCGGATCTAGATAACTGA**TTGGTCATGCCTTTTAAGACC**
	Reverse	AGGTCCGTGCCCCTAAAG
eGFP	Forward	**TGTTGAGTAGGCTTG**ATGGTGAGCAAGGGCGA
	Reverse	**GGTCTTAAAAGGCATGACCAA**TCAGTTATCTAGATCCGGTGGA
Up-eGFP-Down	Forward	GCCTCGAACAGACTAACCG
	Reverse	AGGTCCGTGCCCCTAAAG
Generation of ΔgI ILTV		
Upstream of gI	Forward	GCTCAACCCGATTTCTAACG
	Reverse	CCTCGCCCTTGCTCACCAT**GCTTTTCGAACGTCCCAA**
Downstream of gI	Forward	CGGATCTAGATAACTGA**TTAAGTCTGAATGTGGCTCTCC**
	Reverse	AGGCATCTCTGGTTGGTTG
eGFP	Forward	**TTGGGACGTTCGAAAAGC**ATGGTGAGCAAGGGCGAGG
	Reverse	**GGAGAGCCACATTCAGACTTAA**TCAGTTATCTAGATCCG
Up-eGFP-Down	Forward	GCTCAACCCGATTTCTAACG
	Reverse	AGGCATCTCTGGTTGGTTG
sgRNA-gI	Forward	CACCGACCAGTGCTACCAGG
	Reverse	AAACCCTGGTAGCACTGGTTC
Sequencing sgRNA-plasmid		
PX330	Forward	TGGACTATCATATGCTTACCG
	Reverse	TAGATGTACTGCCAAGTAGGAA
Amplification of recombination regions		
CSW-1 ILTV	Forward	CGCTCTGATATAACAAACCAGTG
	Reverse	CAGCCTTTCCTTCCCTTC
A20 ILTV	Forward	GTTGGCCCGTATAAC
	Reverse	GATCACCGCGGCCAGATCG

*Primer sequences in both bold and standard text format are SOE PCR primers. The different regions of these primers correspond to the different target templates

All the mutants, except for the ΔgI CSW-1 ILTV mutant, were generated using standard homologous recombination techniques. For this, linearized plasmid was transfected into LMH cells along with ILTV genomic DNA and an expression plasmid containing the ILTV ICP4 gene, as previously described [15]. After co-transfection, virus plaques expressing eGFP were identified by fluorescence microscopy. Isolated fluorescent plaques were picked with a micropipette, frozen at  − 70 °C and thawed at 37 °C. The thawed material was used to inoculate LMH and CEK cells grown in six-well plates under an overlay medium of 1% v/v methylcellulose (Sigma-Aldrich) in DMEM to separate recombinant virus (expressing eGFP) from the parent (wildtype) virus by plaque purification. This process was repeated for a minimum of 3 times before further viral characterisation.

To generate the ΔgI CSW-1 ILTV mutant using CRISPR/Cas9-assisted recombination in a transfection/infection system, a single guide RNA (sgRNA) plasmid was generated as previously described [21], with the sgRNA designed to target the open reading frame of the gI gene (Table 1). The construct was verified by Sanger sequencing using the primers in Table 1. For transfection/infection, LMH cells were seeded in 6-well plates and co-transfected with 2 μg each of the repair and CRISPR/Cas9 plasmids using the X-tremeGENE HP DNA transfection reagent (Roche) according to the manufacturer’s instructions. The cells were incubated at 37 °C in a humidified atmosphere of 5% CO_2_ in air for approximately 5 h. CSW-1-ILTV was then added at a multiplicity of infection (moi) of 0.01. After a further 2 h the inoculum and transfection complex were removed and replaced with 2 mL of maintenance medium (DMEM with 1% v/v FBS and 50 µg /ml ampicillin) per well. Incubation was continued for 2–3 days until plaques expressing GFP were observed using fluorescence microscopy. Those plaques were then picked and passaged in LMH and CEK cell cultures under an overlay of 1% v/v methylcellulose medium to separate recombinant virus from the parent virus. This process was repeated for a minimum of 3 times before further viral characterisation.

### In vitro characterisation of fluorescent plaque-purified ILTV

The cytopathic effects (CPE) in infected cells were observed using bright field and fluorescence microscopy. DNA was extracted (QIAEX II, Qiagen) from virus plaques, or from single fluorescent cells, and used as template in PCRs to amplify the expected recombination region for subsequent DNA sequencing. The forward and reverse primers (Table 1) were designed to anneal outside of the expected recombination region and include a small region both upstream and downstream of this region. The 50 µl reaction mixture contained 1.5 mM MgCl_2_, 1.0 unit of Platinum® Taq DNA Polymerase (Invitrogen), PCR Buffer (Invitrogen), 0.2 mM of each dNTP (Promega), 0.1 µM of each primer and 70 ng of template DNA, or sterile water for the contamination control reactions. The reactions were incubated in an iCycler Thermal Cycler (BioRad) at 94 °C for 30 s, followed by 30 cycles of denaturing at 94 °C for 30 s, annealing at 58 °C for 30 s, extension at 72 °C for 4 min, and a final extension step of 72 °C for 10 min. The PCR products were separated and visualized by 0.8% w/v agarose gel electrophoresis and visualised using SYBR Safe DNA Gel Stain in 0.5 × TBE (Thermofisher) and imaged using the ChemiDoc MP imaging system (BioRad).

### DNA sequencing

PCR products were gel-extracted (QIAEX II Gel Extraction Kit, Qiagen) and Sanger sequenced (BDT chemistry version 3.1, Applied Biosystems)) according to the manufacturers’ instructions. The reactions were incubated in an iCycler Thermal Cycler at 95 °C for 5 min, then through 30 cycles of 96 °C for 10 s, 50 °C for 5 s and 60 °C for 4 min. The samples were submitted to the Australian Genome Research Facility (AGRF) for sequence analysis. Sequencing results were analysed using Geneious version 11.1.4 [22].

### Comparison of gD genes in different ILTV strains

It is possible that gD and gI/gE have overlapping functions. To investigate whether the inability of the A20 and CSW-1 derived mutants, but not the A489 derived mutants, to be separated from wildtype virus was related to differences in gD, we compared the predicted protein sequences for gD from CSW-1 ILTV (JX646899), A20 ILTV (JN596963) and A489 ILTV (KY423284.1). These sequences were retrieved from GenBank and aligned and analysed using Geneious 11.1.4

## Results

### Characterisation of ΔgE and ΔgI mutants of CSW-1 ILTV

#### Growth in cell culture

**Fig. 2:**
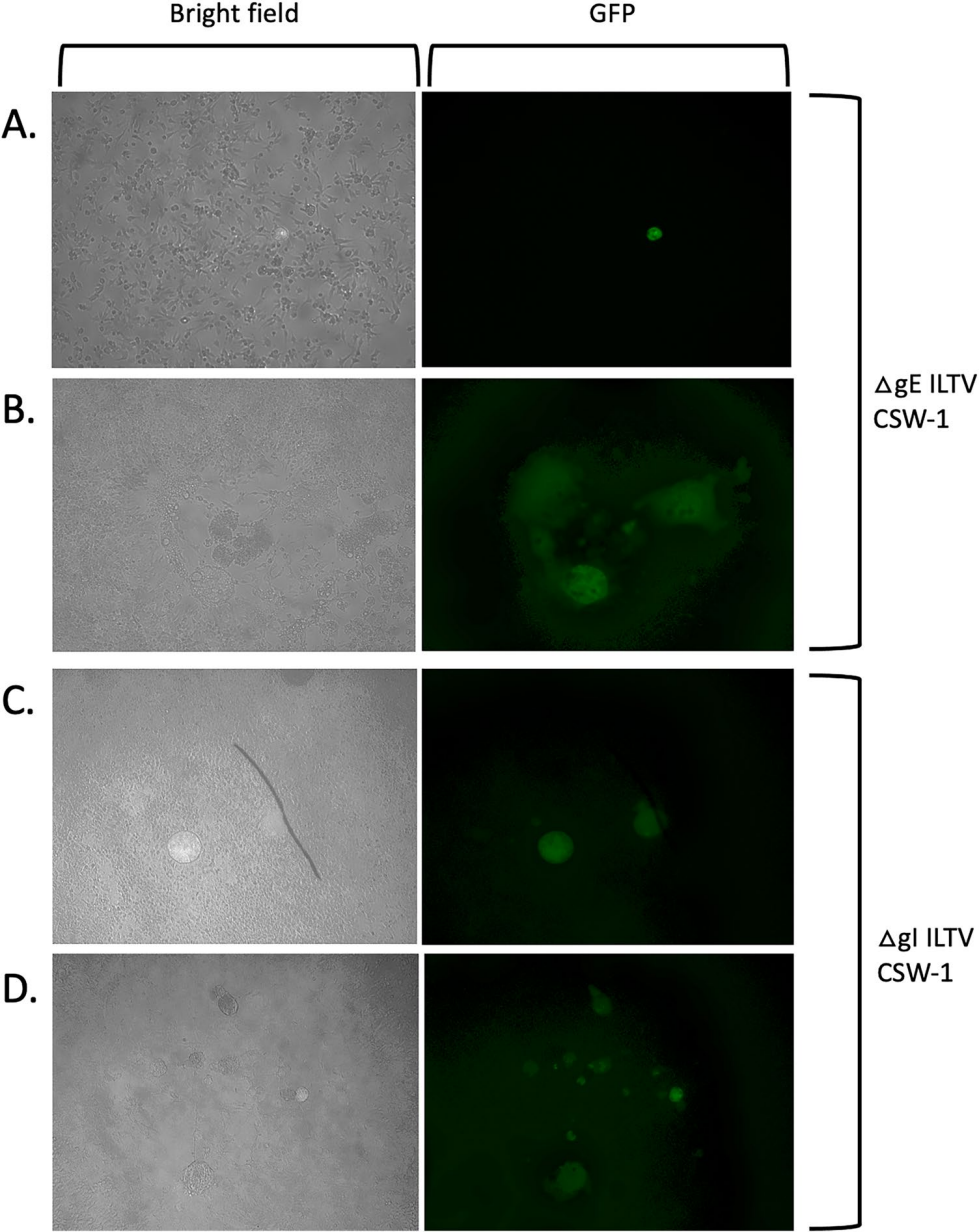
Cytopathic effects induced by CSW-1 ILTV mutants (ΔgE = panels (**A**, **B**), ΔgI = panels (**C**, **D**)) after plaque purification and infection of LMH cells, as observed using bright field and fluorescence microscopy. Two different types of fluorescent CPE were observed: singe round fluorescent cells (panels **A** and **C**) and fluorescent plaques (panels **B** and **D**). Panels **A–D** = 10 × objective. All cultures also contained non-fluorescent plaques with wildtype ILTV morphology (not shown)

**Fig. 3:**
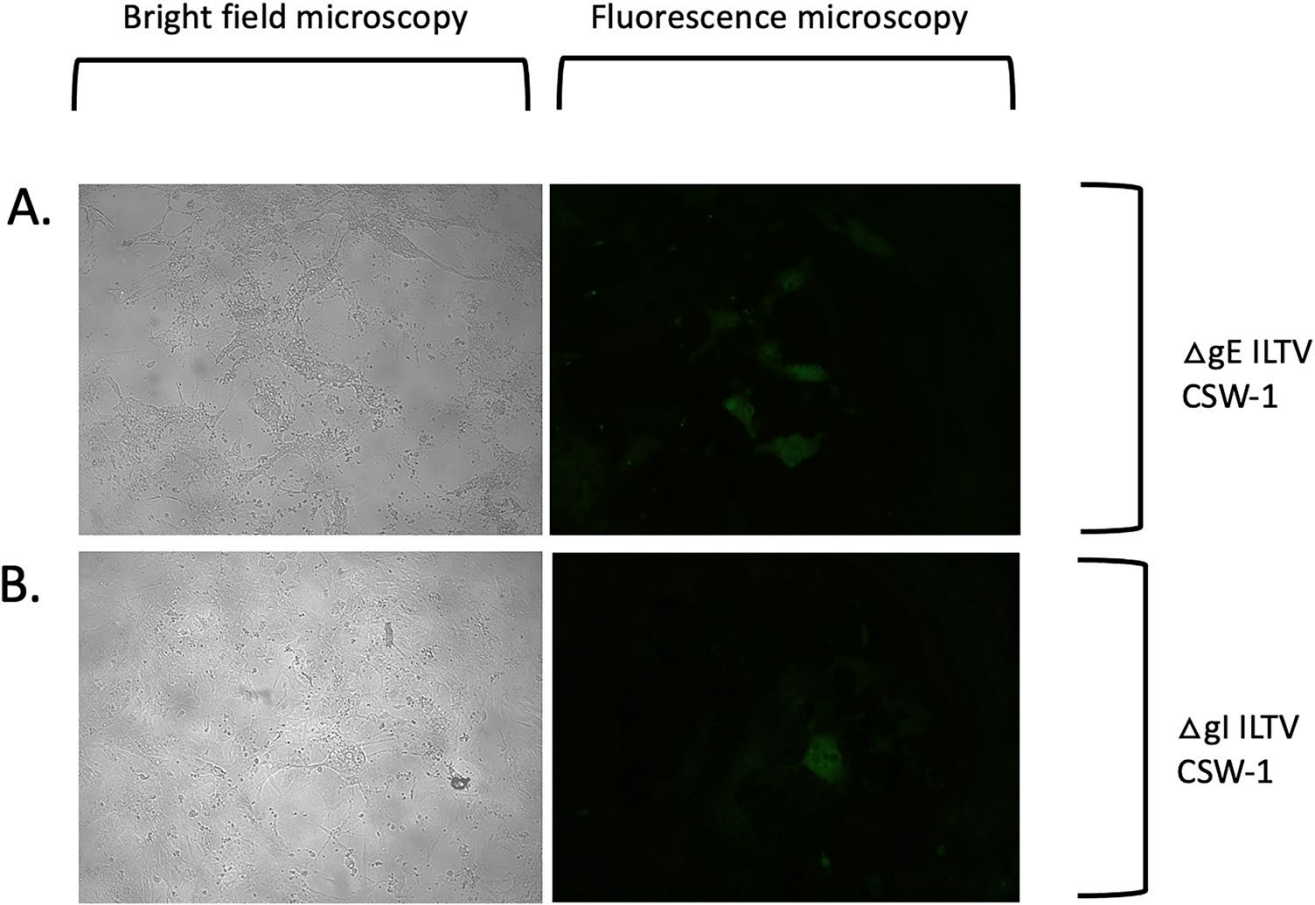
Cytopathic effects induced by CSW-1 ILTV mutants (ΔgE = panel (**A**), ΔgI = panel (**B**)) after plaque purification and infection of CEK cells as observed using bright field and fluorescence microscopy. Panel **A–B** = 10 × objective. All cultures also contained non-fluorescent plaques of wildtype ILTV morphology (not shown)

When the progeny viruses from the CSW-1 co-transfection or infection/transfection procedures that were used to generate ΔgE and ΔgI mutants were plaque purified on LMH cells, two forms of fluorescent CPE were observed in infected LMH cell cultures using fluorescence microscopy. These consisted of i) plaques with wildtype morphology (cleared areas of cell lysis surrounded by enlarged and/or multinucleated cells), or ii) single-infected green cells ((Fig. 2). In addition, non-fluorescent plaques consistent with the CPE expected for parental (wildtype) virus were observed in all cultures. When the same co-transfection or infection/transfection progeny viruses were plaque purified and propagated on CEK cells, similar results were observed (Fig. 3), with all cultures also containing non-fluorescent plaques with the CPE consistent with that expected for parental (wildtype) virus.

### PCR amplification of viral DNA present in the different types of CPE

**Fig. 4:**
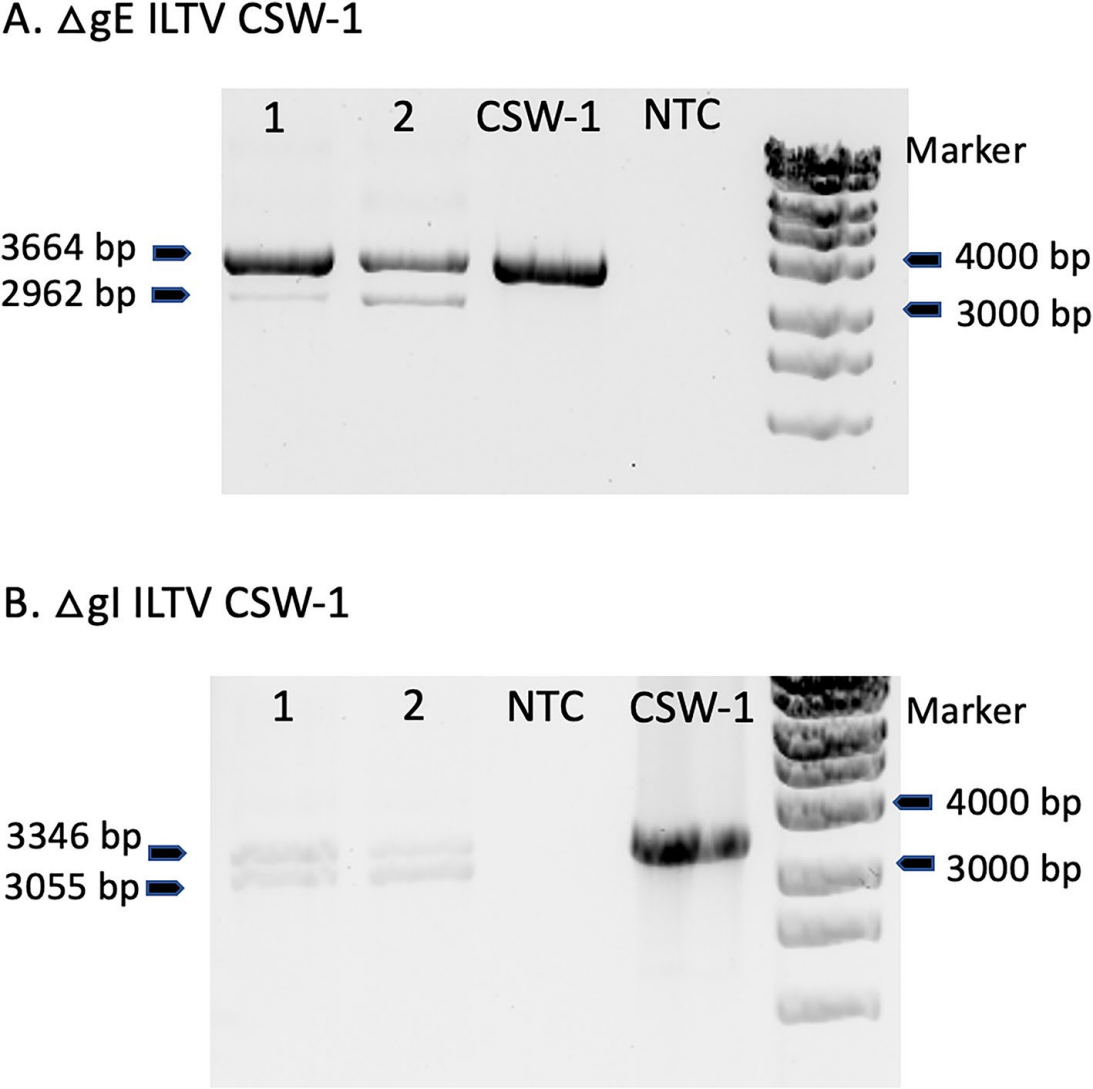
PCR products of viral DNA extracted from fluorescent CPE in LMH cells using plaque-purified progeny viruses from experiments to generate ΔgE (**A**) and ΔgI (**B**) CSW-1 ILTV mutants, each expressing GFP in place of the deleted gene. Sample 1 used extracted DNA from CPE showing the phenotype of single, rounded fluorescent cells. Sample 2 used extracted DNA from fluorescent plaques. CSW-1 ILTV DNA was used as a positive control. The negative control did not have any DNA template (no template control, NTC). The lower intensity of the bands for the ΔgI mutants, compared to the ΔgE mutants, is likely due to a lower quantity of template viral DNA in these samples

PCR amplification of the expected recombination region in the viral DNA present in the different types of fluorescent CPE yielded PCR products of different sizes (Fig. 4). In experiments to generate ΔgE ILTV, a PCR product consistent with amplification of wildtype CSW-1 DNA (3664 bp) was present, as well as a 2962 bp product consistent with the expected size of ΔgE ILTV where the gE coding sequence had been replaced with the GFP coding sequence (2962 bp). In the experiments to generate a ΔgI ILTV a PCR product consistent with the expected size of ΔgI ILTV where the gI coding sequence had been replaced with the GFP coding sequence (3055 bp) was present, in addition to the 3346 bp wildtype CSW-1 product (Fig. 4).

### Sequences of PCR products amplified from virus present in the different types of CPE

Sequence analysis revealed that the DNA sequences of the 3664 and 3345 bp products were identical to those of the wildtype CSW-1 ILTV. Sequence analysis of the 2962 bp product revealed that it had the sequence expected from successful homologous recombination between wildtype ILTV and the gE repair plasmid, with the gE coding region replaced by eGFP. Sequence analysis of the 3055 bp product revealed the sequence that was expected from successful homologous recombination between wildtype ILTV and the gI repair plasmid, with the gI coding region replaced by eGFP. The sequence of the genes adjacent to the deleted gE gene (~ 270 nucleotides of US7 gene and ~ 360 nucleotide of US9 gene) or gI gene (~ 700 nucleotide of US6 gene and ~ 900 nucleotide of US8 gene) remained unchanged. The sequences of the regions immediately upstream and downstream of the recombination region contained no disruptions. These results suggest that, while the gE and gI genes had been successfully deleted, these mutant viruses could not propagate in the absence of wildtype/parental virus.

### Characterisation of ΔgE and ΔgI mutants of A20 ILTV

**Fig. 5:**
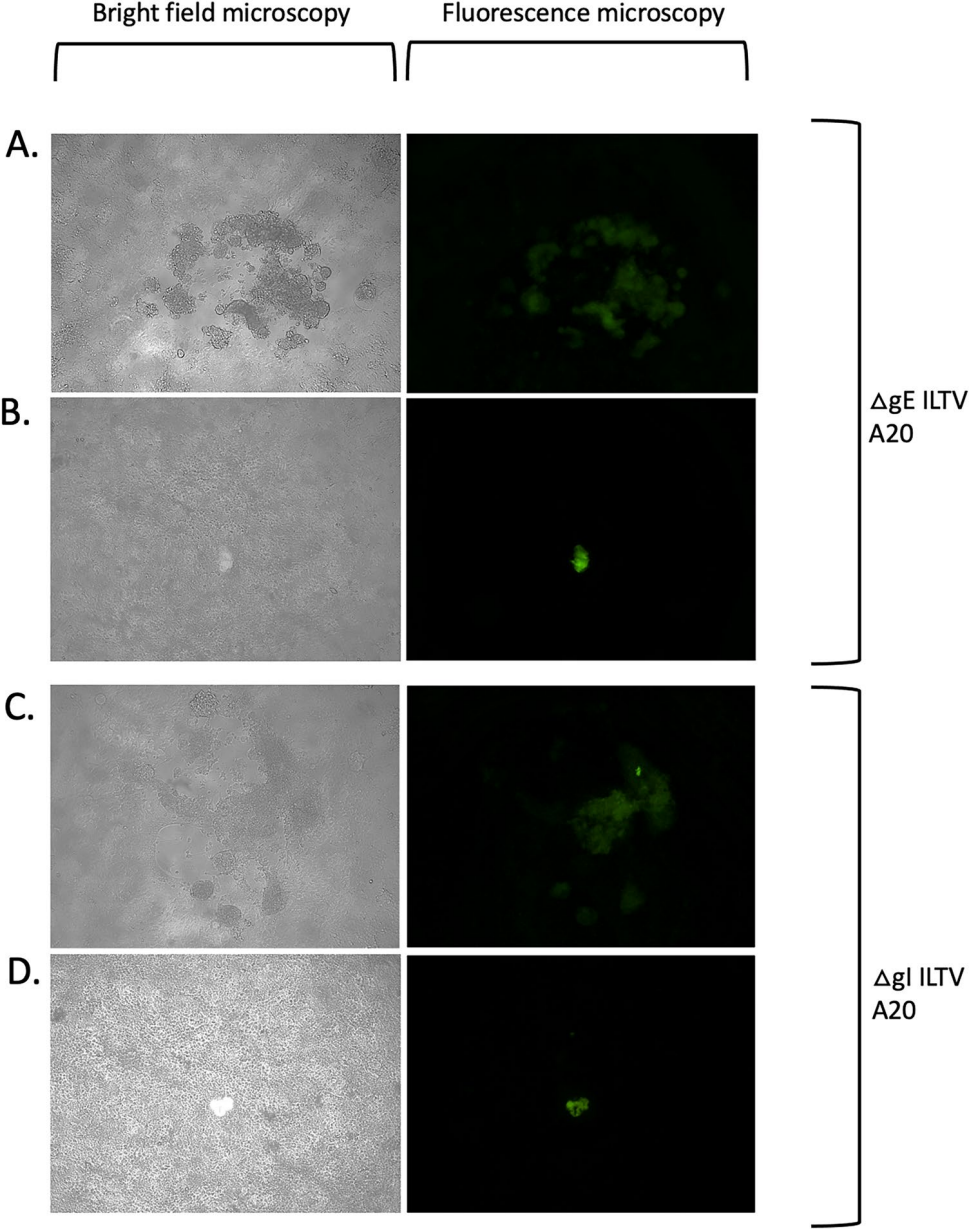
Cytopathic effects induced by A20 ILTV mutants (ΔgE = panels (**A**, **B**), ΔgI = panels (**C**, **D**)) after plaque purification and infection of LMH cells, as observed using bright field and fluorescence microscopy. Two different types of fluorescent CPE were observed; singe round fluorescent cells (panels **B** and **D**) and fluorescent plaques (panels **A** and **C**). Panels **A–D** = 10 × objective. All cultures also contained non-fluorescent plaques with wildtype morphology (not shown)

**Fig. 6:**
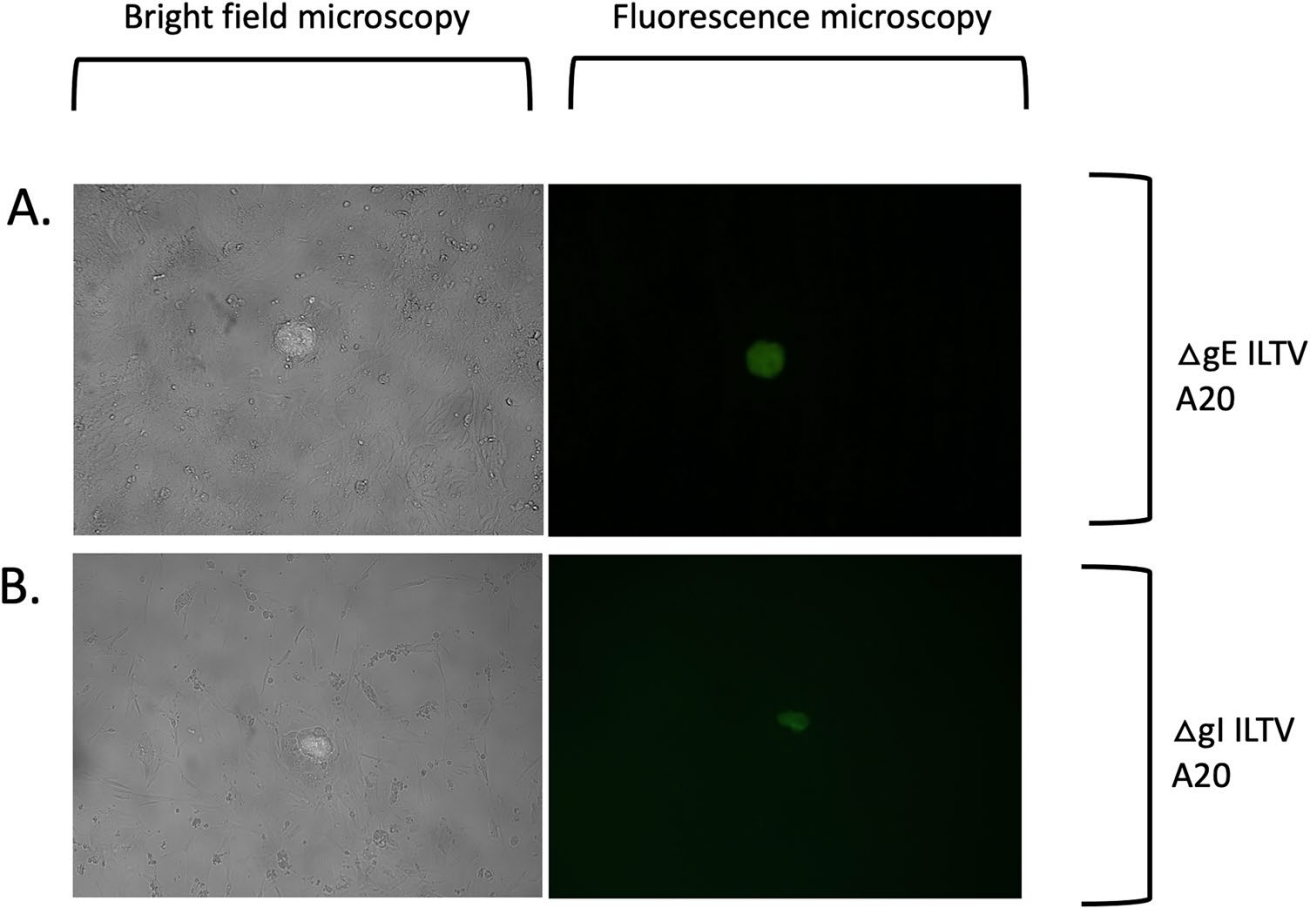
Cytopathic effects induced by A20 ILTV mutants (ΔgE = panel (**A**), ΔgI = panel (**B**)) after plaque purification and infection of CEK cells, as observed using bright field and fluorescence microscopy. Panels **A–B**  = 10 × objective. All cultures also contained non-fluorescent plaques with wildtype morphology (not shown)

As attempts to generate and propagate pure cultures of ΔgE and ΔgI mutants of CSW-1 ILTV were unsuccessful (the mutants could not be cultured separately from wildtype virus), the same procedures were repeated using a different ILTV strain (A20 ILTV) [23, 24]. When the progeny viruses from the A20 co-transfection procedures to generate ΔgE and ΔgI mutants were plaque purified on either LMH cells or CEK cells, fluorescent CPE similar to that seen with the CSW-1 mutants was seen (Figs. 5 and 6). In addition to the similar patterns of fluorescence, all progeny virus cultures also contained non-fluorescent plaques consistent with the CPE expected from parent (wildtype) virus.

### Comparison of the gD coding sequences in Australian ILTV strains and A489 ILTV

In order to investigate whether the inability of ILTV CSW-1 and A20 ΔgE and ΔgI mutants to be separated from wildtype virus may be linked to differences in their gD (as it is possible that gD and gI/gE have overlapping functions) the gD gene sequences from these strains were compared with that of the ILTV A489 strain. Alignment of the predicted protein sequences of gD from CSW-1, A20 and A489 ILTV revealed minimal differences. There were only five differences (all single amino acid replacements) that were consistent between the two Australian strains (CSW-1 and A20 ILTV) and A489 ILTV. There were no differences in key structural regions of gD, including the cysteine residues that form the structurally important intramolecular disulphide bonds, or the N-linked glycosylation sites (Supplementary Fig. 1).

## Discussion

The gE and gI deletion mutants of ILTV generated in this study were unable to be separated from wildtype virus using different cell lines and multiple plaque purification steps. This was similar to results from a previous study conducted in our laboratory of a gI/gE double deletion mutant of CSW-1 [25] and also similar to findings with gE/gI deletion mutants of MDV and VZV [9–11, 15]. However, this contrasts with findings from studies of gE/gI deletion mutants of most other alphaherpesviruses (7, 8), including those of a 2013 study in which gE and gI were shown to be non-essential for ILTV replication in vitro using the ILTV strain A489 [16].

The reasons for the different findings in different studies of ILTV gI and gE deletion mutants are unclear. The essential nature of gE and gI in MDV and VZV appear to be associated with an absence of gD, either because the gD gene is absent from the viral genome (VZV) [12] or because gD is not expressed in cell culture (MDV) [13]. We compared the predicted protein sequences derived from the gD genes of the A20, CSW-1 and A489 ILTV strains, but a high level of sequence identity was seen. Previous studies have shown that gD is expressed in CSW-1, SA2 (an Australian vaccine strain from which the closely related A20 ILTV vaccine strain was derived) and A489 ILTV strains propagated in cell culture [16, 26–28]. These findings suggest that there are no notable differences in gD sequence or expression between the different ILTV strains. Thus, it is unlikely that the apparent differences in the importance of gI and gE for in vitro viral replication in these ILTV strains are due to differences in the role or function of gD.

VZV and MDV are highly cell-associated viruses, with little or no infectious virus released into the supernatant of infected cultured cells [29–33]. ILTV is not considered to be a highly cell-associated virus, but it is possible that there are some differences between ILTV strains in their level of cell association, and that the Australian CSW-1 and A20 ILTV strains could be more highly cell associated than the A489 strain. Whether this could be linked to a higher reliance on gI and gE for cell-to-cell spread and thus propagation in cell culture is not known, but further work to compare the level of cell association, the level of production of gD and the essential nature of gE and gI in different ILTV strains would help to clarify this.

The possibility of off-target disruptions in the genomes of the gI and gE deleted viruses generated in this study should also be considered. This was proposed as a possible mechanism underlying the phenotype of the original gI/gE double deletion mutant of CSW-1 [16], but would be unlikely to have occurred multiple times in two different virus strains and across four different, independently derived mutants. Only the genome regions immediately upstream and downstream of the recombination regions were sequenced in this study. The inability to propagate the virus in cell culture without wildtype virus present prevented further sequencing of the genomes of the mutant viruses.

For both the ΔgE and ΔgI CSW-1 mutants, the single-infected fluorescent cells, and the fluorescent plaques both contained a mixture of DNA from deletion mutant viruses and wildtype virus. This differs slightly from the results presented in our previous attempt to produce a double gE and gI deletion mutant of CSW-1, where DNA from just the deletion mutant virus could be detected in single, fluorescent cells [25]. The mixture of wildtype and mutant virus DNA detected in single-infected cells in this study could reflect contamination with wildtype viruses, which were abundant in the cultures despite repeated attempts to plaque purify the mutant virus away from wildtype virus.

In this study we used the CRISPR/Cas9-assisted homologous recombination and transfection/infection system to generate a ΔgI CSW-1 ILTV mutant. This was performed to determine if CRISPR/Cas9-assisted homologous recombination could help us to obtain a pure culture of the deletion mutant, without wildtype virus present. Application of CRISPR/Cas9 mutagenesis to the ILTV genome has been described previously in the context of generating vectored vaccines [34] however, this current study is the first to use CRISPR/Cas9 to study gene function in ILTV. Despite the potential advantages of this approach, which can reduce the proportion of wildtype viruses and increase the proportion of mutant viruses after transfection/infection [35, 36], in this study it was insufficient to allow purification of ΔgI CSW-1 ILTV away from wildtype viruses. In future studies it would be helpful to compare CRISPR-Cas9-assisted and traditional homologous recombination approaches when targeting ILTV genes for deletion that aren’t so important for viral replication in cell culture. This may give a better indication of the usefulness of CRISPR-Cas9-assisted homologous recombination for generating different ILTV deletion mutants as it is likely that the advantages may be more apparent when such genes are targeted. As suggested in our previous study [25], a bacterial artificial chromosome (BAC) ILTV may be a useful tool for studying the roles of ILTV genes that are essential or important for viral replication. More recently, codon pair bias deoptimization has been successfully applied to study essential genes of MDV [37] and could be applied to ILTV to help characterise essential or important genes associated with viral replication in vitro.

## Supplementary Information

Below is the link to the electronic supplementary material.

Supplementary file1 (DOCX 403 kb)
