# Supplementary material for: Construction and characterisation of glycoprotein E and glycoprotein I deficient mutants of Australian strains of infectious laryngotracheitis virus using traditional and CRISPR/Cas9-assisted homologous recombination techniques
Source: Virus Genes. 2022 Sep 20;58(6):540–9. doi: 10.1007/s11262-022-01933-5 (PMC9636094; doi:10.1007/s11262-022-01933-5)
Supplement: Supplementary file 1 — Supplementary file1 (DOCX 403 kb) [file 11262_2022_1933_MOESM1_ESM.docx]

**Supplementary information**

Construction and characterisation of glycoprotein E and glycoprotein I deficient mutants of Australian strains of infectious laryngotracheitis virus using traditional and CRISPR/Cas9-assisted homologous recombination techniques

Marzieh Armat ^1^, Paola K Vaz ^1^, Glenn F Browning ^1^, Amir H Noormohammadi ^2^, Carol A Hartley ^1^^, & Joanne M Devlin ^1^*^

1-Asia-Pacific Centre for Animal Health, Department of Veterinary Biosciences, Melbourne Veterinary School, Faculty of Veterinary and Agricultural Sciences, University of Melbourne, Parkville, Victoria, Australia

2-Asia-Pacific Centre for Animal Health, Faculty of Veterinary and Agricultural Sciences, The University of Melbourne, Werribee, Victoria, Australia

^ These authors contributed equally

*Corresponding Author: [devlinj@unimelb.edu.au](mailto:devlinj@unimelb.edu.au)

**Supplementary Fig. 1**


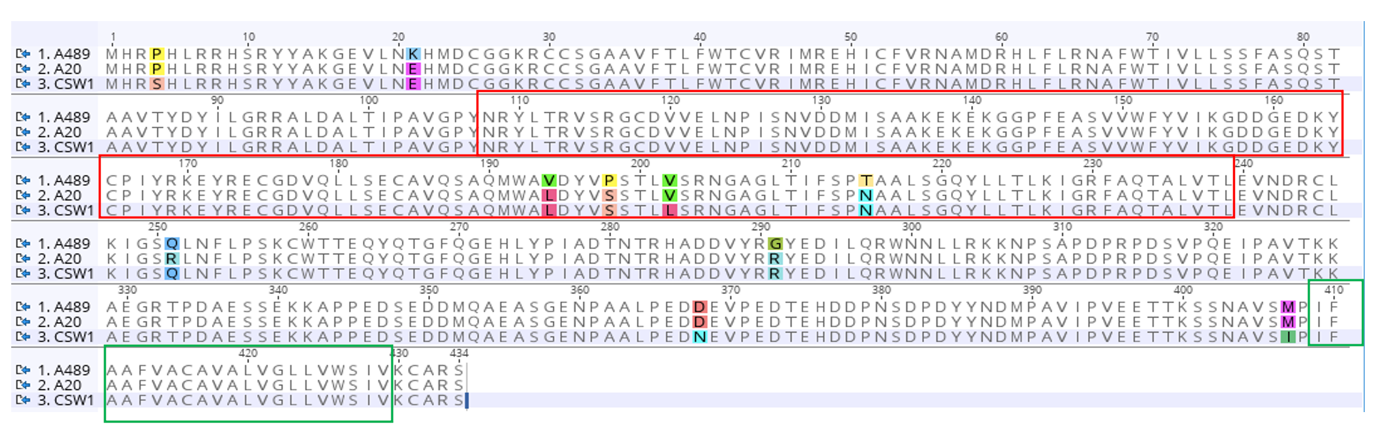


Schematic showing the alignment of the predicted protein sequences of A489, A20 and CSW-1 ILTV glycoprotein D. The predicted transmembrane region (aa 409 to 429) is boxed in green. The red box contains the region of the conserved protein domain family "Herpesvirus glycoprotein D/GG/GX" (pf01537) (aa 108 to 239)
